# Increasing NPYergic transmission in the hippocampus rescues aging-related deficits of long-term potentiation in the mouse dentate gyrus

**DOI:** 10.3389/fnagi.2023.1283581

**Published:** 2023-11-09

**Authors:** Katharina Klinger, Miguel del Ángel, Gürsel Çalışkan, Oliver Stork

**Affiliations:** ^1^Department of Genetics and Molecular Neurobiology, Institute of Biology, Otto-von-Guericke University, Magdeburg, Germany; ^2^Research Group “Synapto-Oscillopathies”, Institute of Biology, Otto-von-Guericke-University, Magdeburg, Germany; ^3^Center for Behavioral Brain Sciences, Magdeburg, Germany; ^4^Center for Intervention and Research on Adaptive and Maladaptive Brain Circuits Underlying Mental Health (C-I-R-C), Magdeburg, Germany; ^5^German Center for Mental Health (DZPG), Magdeburg, Germany

**Keywords:** healthy aging, dorsal dentate gyrus, long-term potentiation, neuropeptide-Y, cholinergic system, NPYergic system

## Abstract

Loss of neuropeptide Y (NPY)-expressing interneurons in the hippocampus and decaying cholinergic neuromodulation are thought to contribute to impaired cognitive function during aging. However, the interaction of these two neuromodulatory systems in maintaining hippocampal synaptic plasticity during healthy aging has not been explored so far. Here we report profound sex differences in the Neuropeptide-Y (NPY) levels in the dorsal dentate gyrus (DG) with higher NPY concentrations in the male mice compared to their female counterparts and a reduction of NPY levels during aging specifically in males. This change in aged males is accompanied by a deficit in theta burst-induced long-term potentiation (LTP) in the medial perforant path-to-dorsal DG (MPP-DG) synapse, which can be rescued by enhancing cholinergic activation with the acetylcholine esterase blocker, physostigmine. Importantly, NPYergic transmission is required for this rescue of LTP. Moreover, exogenous NPY application alone is sufficient to recover LTP induction in aged male mice, even in the absence of the cholinergic stimulator. Together, our results suggest that in male mice NPYergic neurotransmission is a critical factor for maintaining dorsal DG LTP during aging.

## 1. Introduction

Neuronal hyperactivity and reduced capacity of synapses to express long-term plasticity are commonly observed in neurodegenerative diseases (Bakker et al., 2012; Seo et al., 2021) and during aging (Barnes and Mcnaughton, 1980; Geinisman et al., 1995). The hippocampus, one of the hub regions that play a central role in learning and memory (Squire and Zola, 1996), shows such hyperactivity and aberrant synaptic plasticity (Barnes and Mcnaughton, 1980; Geinisman et al., 1995). Therefore, studies that aim at identifying treatment strategies for normalizing hippocampal function are of utmost importance to mitigate cognitive decline during aging.

Neuropeptide-Y (NPY) belongs to the pancreatic polypeptides class and is widely distributed in the nervous system (Armstrong et al., 1982). NPY is highly expressed in the hilus of the dentate gyrus (DG) (Freund and Buzsáki, 1996; Raza et al., 2017), the gate of the hippocampal trisynaptic circuit that plays a pivotal role in encoding contextual and spatial information (Ramirez et al., 2013; Raza et al., 2017). The DG receives excitatory perforant path (PP) projections from the entorhinal cortex that are substantially affected in the aging process in rodents (Amani et al., 2021) and humans (Yassa et al., 2010). We and others have previously shown that NPY plays a crucial role in controlling DG circuit function and PP-induced physiological responses in young adult mice (Madronal et al., 2016; Raza et al., 2017). Importantly, a substantial decline in the levels of NPY and NPY receptor type 1 (Y1-R), which is highly expressed in DG excitatory granule cells (GC), has been shown during the aging process (Hattiangady et al., 2005; Botelho and Cavadas, 2015). However, to the best of our knowledge, no studies have yet systematically investigated the mechanistic involvement of NPYergic transmission in the PP-induced neurotransmission and plasticity in the DG of aged mice.

Disturbances of the hippocampal circuit function during aging are accompanied by aberrant functional or structural alterations in the long-range cholinergic projections (e.g., synaptic loss, cholinergic axonal degeneration) from the medial septum to the hippocampus (Kesner et al., 1986; Ballinger et al., 2016) and are frequently used to model age–related changes in hippocampal function (Berger-Sweeney et al., 2001). Accordingly, the beneficial effects of acetylcholine esterase (AChE) inhibitors for the treatment of Alzheimer’s Disease and on long-term plasticity in aged rodents are well documented (Pang et al., 1993; Yiannopoulou and Papageorgiou, 2020). It is important to note that cholinergic modulation of NPYergic neurotransmission is critically involved in the modulation of DG circuit function and DG-dependent behaviors (Baratta et al., 2002; Raza et al., 2017). Specifically, enhanced cholinergic drive leads to Y1-R-dependent NPYergic signaling and altered DG output via activation of the hilar Somatostatin (SST) containing interneurons (Raza et al., 2017), most of which co-express NPY (Freund and Buzsáki, 1996; Houser, 2007). Intriguingly, to date, the potential use of such synergistic interactions between the cholinergic and NPYergic systems in sustaining hippocampal DG neurotransmission and plasticity during aging has not been systematically explored.

Therefore, we addressed the potential involvement of NPY in mediating cholinergic modulation of plasticity in the aging hippocampus and explored the possibility that enhancement of NPYergic signaling may provide a resource for alleviating age-induced synaptic transmission and plasticity changes. It needs to be considered that both the cholinergic and the NPYergic system display remarkable sex differences. Particularly, the reduction in the density of cholinergic fibers targeting the DG is significantly larger in aged male rats than in aged females (Lukoyanov et al., 1999). Moreover, NPY levels differ between sexes in the hippocampus, with higher NPY levels in young males compared to female counterparts (Nahvi and Sabban, 2020). Based on the decay of NPY expression that we observed specifically in the dorsal DG of aged (>20-month-old) male mice, we collected evidence that NPYergic signaling is required for the cholinergic (via physostigmine) alleviation of aging-related LTP deficits and that exogenous NPY application is sufficient to ameliorate the LTP deficit in these animals.

## 2. Methods

### 2.1. Animals

Wild-type male and female C57BL/6 mice (C57BL/6BomTac; M&B Taconic, Germany) were housed and bred in the animal facility of the Department of Genetics and Molecular Neurobiology, Institute of Biology, Otto von Guericke University Magdeburg under standard laboratory conditions. Young-adult (2–4 months) and aged (>20 months) males and females were kept in groups of two to five individuals in an inverse 12 h light/ dark cycle (lights on from 7 PM – 7 AM with a 30 min dawn phase). They had access to food and water *ad libitum*. All experiment preparations were conducted during the animals’ dark (active) phase, between 8 AM and 3 PM.

### 2.2. Electrophysiology

#### 2.2.1. Slice preparation

To investigate synaptic plasticity and transmission during aging and under different pharmacological conditions, young-adult and aged mice of both sexes were firstly deeply anesthetized with isoflurane and then decapitated. Brains were rapidly (within ~60 s) removed and placed into cold (4–8°C) carbonated (5% CO2 / 95% O2) artificial cerebrospinal fluid (aCSF) containing (in mM) 129 NaCl, 21 NaHCO3, 3 KCl, 1.6 CaCl2, 1.8 MgSO4, 1.25 NaH2PO4, and 10 glucose. Parasagittal slices containing the dorsal hippocampus were obtained by cutting the brain at an angle of about 12° on an angled platform. The four most dorsal slices were transferred to an interface chamber perfused with aCSF at 32 ± 0.5°C (flow rate: 1.8 mL ± 0.2 mL per min, pH 7.4, osmolarity ~300 mosmol kg^−1^). After cutting, the slices were left to rest for 1 h before starting the recordings.

#### 2.2.2. Field potential recordings

For dorsal DG electrophysiology, one glass electrode, filled with aCSF (~ 1 MΩ), was placed at 70–100 μm depth into the mid-molecular cell layer (ML). The stimulation of the medial PP (MPP) was performed with a bipolar tungsten wire electrode, with exposed tips of ~20 μm and tip separations of ~75 μm (electrode resistance in aCSF: ~ 0.1 MΩ, world precision instruments (WPI); Friedberg, Germany). A second bipolar tungsten electrode (same parameters as in the MPP) was placed into the hilus of the dorsal DG to elicit an antidromic stimulation during LTP induction. An input–output (I-O) curve for each stimulation electrode was recorded after stabilization of the responses (0.033 Hz, pulse duration: 100 μs) over 20 min. The baseline excitability and maximal synaptic response were measured by obtaining the I-O curve using pulses with stimulation intensities ranging from 10 to 75 μA. The stimulus intensity that resulted in ~50% of the maximum field excitatory post-synaptic potentials (fEPSP) amplitude of the ML after orthodromic stimulation was subsequently used for baseline recordings with orthodromic stimulation. While the stimulus intensity that resulted in ~70% of the maximum fEPSP amplitude of the ML after antidromic stimulation was used for the antidromic stimulation during LTP induction. The appropriate placement of the orthodromic stimulation was verified through paired-pulse stimulation with different inter-pulse intervals right after obtaining the Input–output (I-O) curve before starting the baseline recording. The characteristic feature of the MPP-DG synapse is the consistent paired-pulse depression at 50 ms inter-pulse interval (Barnes and Mcnaughton, 1980; Froc et al., 2003). In all protocols, baseline and post-theta-burst stimulation (TBS) recordings were done with orthodromic stimulation (in the MPP). Exclusively, the four repetitions repeated at 0.1 Hz of TBS (one burst consisting of 10 pulses at 100 Hz – repeated 10 times at 5 Hz) (Lopez-Rojas et al., 2016) were done with orthodromic followed by antidromic stimulation (in the hilus of the DG). All recordings were performed without GABA blockers to understand the impact of NPY expressing/releasing GABAergic interneurons on LTP in aged animals. Signals were pre-amplified using a custom-made or EXT20-F amplifier (npi electronics, Tamm, Germany) and low-pass filtered at 3 kHz. Signals were sampled at a frequency of 10 kHz and stored on a computer hard disc for offline analysis. The fEPSP slopes (20–80%) were analyzed offline using self-written MATLAB-based analysis tools (MathWorks, Natick, MA, United States).

#### 2.2.3. Pharmacological interventions during electrophysiological recordings

The effect of selective Y1-R blockade and NPY on the fEPSP slope at the MPP-DG synapse during neurotransmission and LTP responses in young and aged males were evaluated in two different experiments. Either the selective Y1-R antagonist BIBP3226 (1 μM; Cat.-No. 2707; Tocris, Bristol, United Kingdom) or NPY (1 μM; Cat.-No. 90880–35-6; Cayman Chemicals, Ann Arbor, Michigan, USA) was added to the aCSF for 20 min or 40 min, respectively, (until TBS) after 20 min baseline recording with aCSF. To evaluate the effect of constitutive cholinergic activation and selective Y1-R blockade under such cholinergic activation on baseline transmission and LTP, the AChE inhibitor physostigmine hemisulfate (PHY; 2 μM; Cat.-No. sc-203661; Santa Cruz Biotechnology, Dallas, Texas, USA) or PHY combined with BIBP3226 (1 μM) was perfused in slices of young and aged male mice. PHY was applied for 60 min or 40 min, followed by 20 min BIBP3226 (1 μM) application after an initial 20 min baseline recording with aCSF. In all experiments, the perfusion solution was changed back to aCSF at the time point of TBS.

### 2.3. Protein analysis

Mice were deeply anesthetized with isoflurane and killed by cervical dislocation. After cervical dislocation, the mice were decapitated, and the brains were carefully removed from the skull and transferred to ice-cold phosphate buffer saline. For protein analysis, the dorsal DG was dissected manually (Hagihara et al., 2010). Samples were snap-frozen with liquid nitrogen and stored at −80°C.

#### 2.3.1. Western blot (WB)

Hippocampal tissue (dorsal DG) of aged and young males and females was mechanically homogenized on ice in cold Laurylmaltoside-buffer containing 1% Laurylmaltoside, 1% NP-40, 1 mM Na_3_VO_4_, 2 mM EDTA, 50 mM Tris–HCl pH 8.0, 150 mM NaCl, 0.5% deoxycholate, 1 mM NaF 1 mM AEBSF protease inhibitor (cat. No. 78431; Thermo Fisher Scientific, Massachusetts, United States), 1 μM Pepstatin A (cat. No. 78436; Thermo Fisher Scientific, Massachusetts, United States), and 1 Tablet of Pierce protease inhibitor (cat. No. A32963; Thermo Fisher Scientific, Massachusetts, USA). The protein concentration was quantified with the RC DC Protein assay Kit II (cat. No. 5000122; Bio-Rad Labortaories Inc., California, United States). After protein concentration assessment, the samples were prepared for immunoblot analysis by adding sample buffer (10% glycerol, 60 mM Tris HCL pH 6.8, 2% SDS, 0.01% Bromophenol blue, and 1.25% mercaptoethanol). For electrophoretic separation, an SDS Bis-Acrylamide gel was loaded with 20 μg of protein from each sample and a standard protein marker (PageRuler Plus, cat. No. 11852124; Thermo Fisher Scientific, United States). The proteins were transferred to FL- PVDF membranes (cat. No. IPFL00010; Merck Millipore; Massachusetts, United States). The membranes were incubated with ms anti-Y1-R (1:500; Santa Cruz Biotechnology, Texas, United States, cat. No. #sc-393192) primary antibody solution at 4°C overnight. A near-infrared labeled secondary antibody IRDye 800CW or IRDye680CW (1:10,000; LI-COR Biosciences, Nebraska, United States) was used to detect the primary antibody. Finally, the imaging of the antibodies was performed using the Odyssey Imaging system (LI-COR Biosciences, Nebraska, United States), and the protein signal was quantified in the Image Studio software (LI-COR Biosciences, Nebraska, United States).

#### 2.3.2. Enzyme-linked immunosorbent assay

To determine the NPY peptide levels, 5 μL of the protein extract from each sample was prepared in a final concentration of 1 μg/μL and analyzed with the ELISA kit for NPY (Product No. CEA879Hu; UOM: 96 T; with a minimum detectable dose less than 9.44 pg./mL) according to manufacturer’s instructions (cloude-clone corp., Texas, United States).

### 2.4. Statistics

Data are reported as mean ± standard error of the mean (SEM). Before the statistical comparison of electrophysiological data, outliers were identified after ROUT, and normality was evaluated with the D’Agostino-Pearson test for all statistical tests. Baseline transmission data was normalized to aCSF condition (10 min before application of the drug of interest), whereas LTP data were normalized to the data obtained 10 min prior to TBS. An in-slice comparison using either a one-tailed paired *t*-test or Wilcoxon signed-rank test (depending on normal distribution) was performed to determine successful LTP induction [as reported previously (Gilbert et al., 2000; Rodgers et al., 2015; Figueroa et al., 2021)]. Furthermore, to evaluate statistical differences across different groups (e.g., LTP strength, pharmacological treatment, or I-O curves), either a two-tailed unpaired t-test, Mann–Whitney *U*-test, or two-way ANOVA, where appropriate, was performed.

ELISA and WB data were analyzed with a two-way ANOVA Geisser–Greenhouse correction and Fisher’s LSD post-hoc test. Graphs and statistical tests were conducted with GraphPad Prism (version 9.4.1(681); Dotmatics, Boston, Massachusetts, United States). Note that *n* accounts for the number of slices while *N* accounts for the number of animals.

## 3. Results

### 3.1. NPY concentration shows a sex- and age-dependent decrease

**Figure 1:**
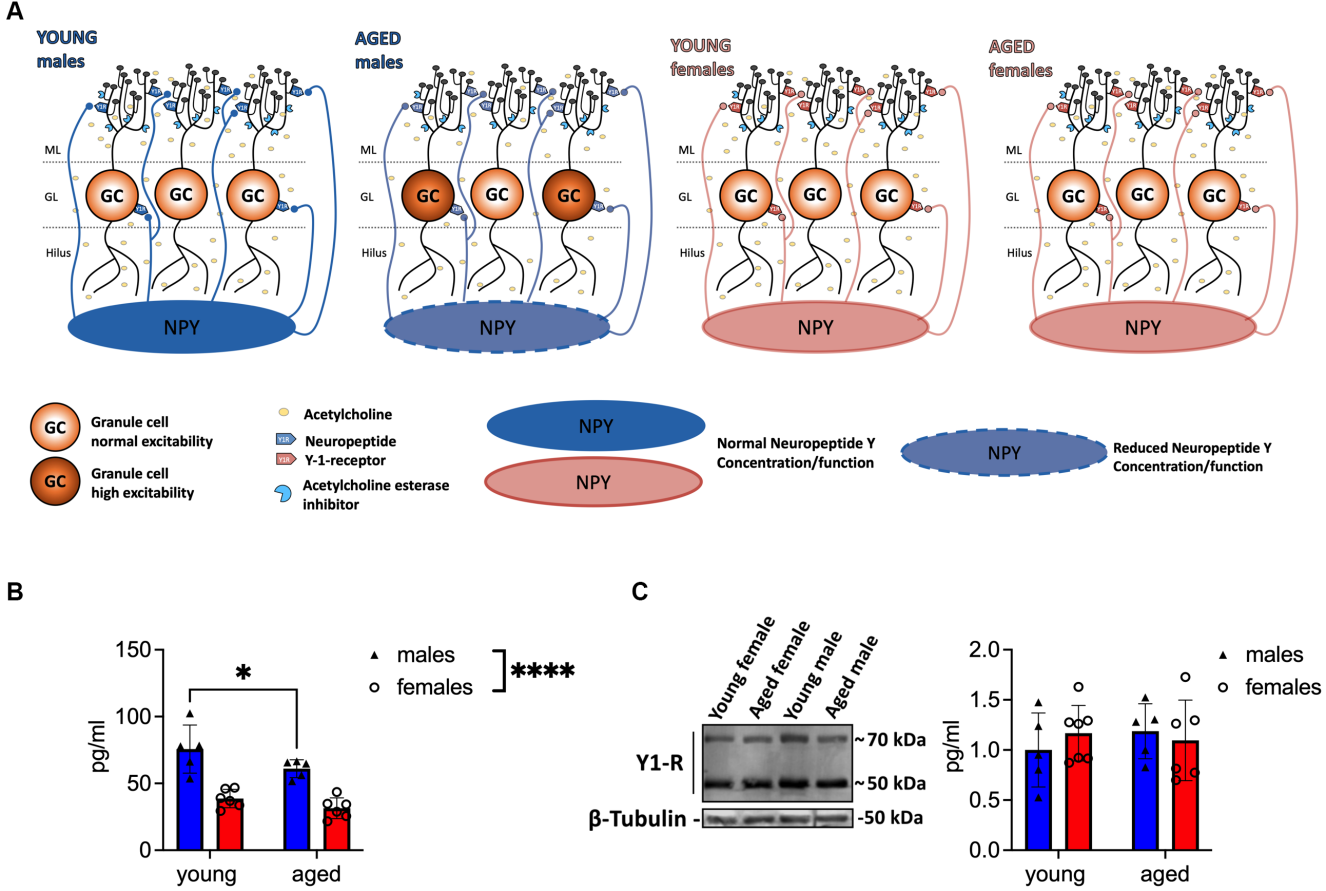
Aged male mice show reduced NPY concentration in the DG. **(A)** Scheme illustrating the reduction of NPY expression in the local circuitry. Reduced levels of NPY are evident in aged male mice, whereas Y1-R expression remains unchanged during aging. The well-established reduction of ACh is indicated with light yellow circles. **(B)** Tissue concentration of NPY in the DG decreases in males during aging. Female mice express low but unaltered NPY levels. **(C)** Y1-R expression measured by immunoblotting does not differ between sexes or over lifetime. A representative WB membrane with one example of each sex and age is presented. ACh, acetylcholine. Changes in NPY concentration (group comparison): *p < 0.05, ****p < 0.0001.

Several studies report a decline in NPY secretion, NPY mRNA, Y1-R, and NPY immunoreactivity during aging (Iri Geinisman et al., 1995; Matsuoka et al., 1995; Hattiangady et al., 2005; Botelho and Cavadas, 2015). Therefore, we investigated potential age-mediated changes in NPY concentration and Y1-R expression on the DG tissue of young-adult and aged male and female mice. In agreement with previous findings (Nahvi and Sabban, 2020), NPY concentration in the DG was significantly lower in females than in males (*F*_(1, 18)_ = 54.08, *p* < 0.0001, two-way ANOVA; post-hoc comparison: young-adult females vs. young-adult males: *p* < 0.0001, Fisher’s LSD test; Figure 1B). This sex difference persisted through aging (post-hoc comparison: aged females vs. young-adult males: *p* < 0.0001; young-adult females vs. aged males: *p* = 0.0026; aged females vs. aged males: *p* = 0.0002, Fisher’s LSD test; *N* = 5 (young-adult males), *N* = 5 (aged males); *N* = 6 (young-adult females); *N* = 6 (aged females); Figure 1B). Strikingly, aged male mice showed reduced NPY levels in comparison to young-adult male mice (*F*_(1, 18)_ = 5.851, *p* = 0.0264, two-way ANOVA; post-hoc comparison *p* = 0.0412, Fisher’s LSD test; Figure 1B), while NPY concentration in females remained unchanged during aging (post-hoc comparison young-adult vs. aged anestrus females: *p* = 0.2537; Figure 1B). No age-dependent changes in Y1-R expression were evident (sex: *F*_(1, 19)_ = 0.1691, *p* = 0.6855; age: *F*_(1, 19)_ = 0.07256, *p* = 0.7906; interaction: *F*_(1, 19)_ = 0.8460, *p* = 0.3692; two-way ANOVA, *n* = 5 (young-adult males); *N* = 5 (aged males); *N* = 7 (young-adult females); *N* = 6 (aged females); Figure 1C) in the DG. To summarize, only aged male mice showed reduced NPY levels in the DG compared to young-adult mice.

### 3.2. LTP induction at the MPP-DG synapse shows sex differences in an age-dependent manner

**Figure 2:**
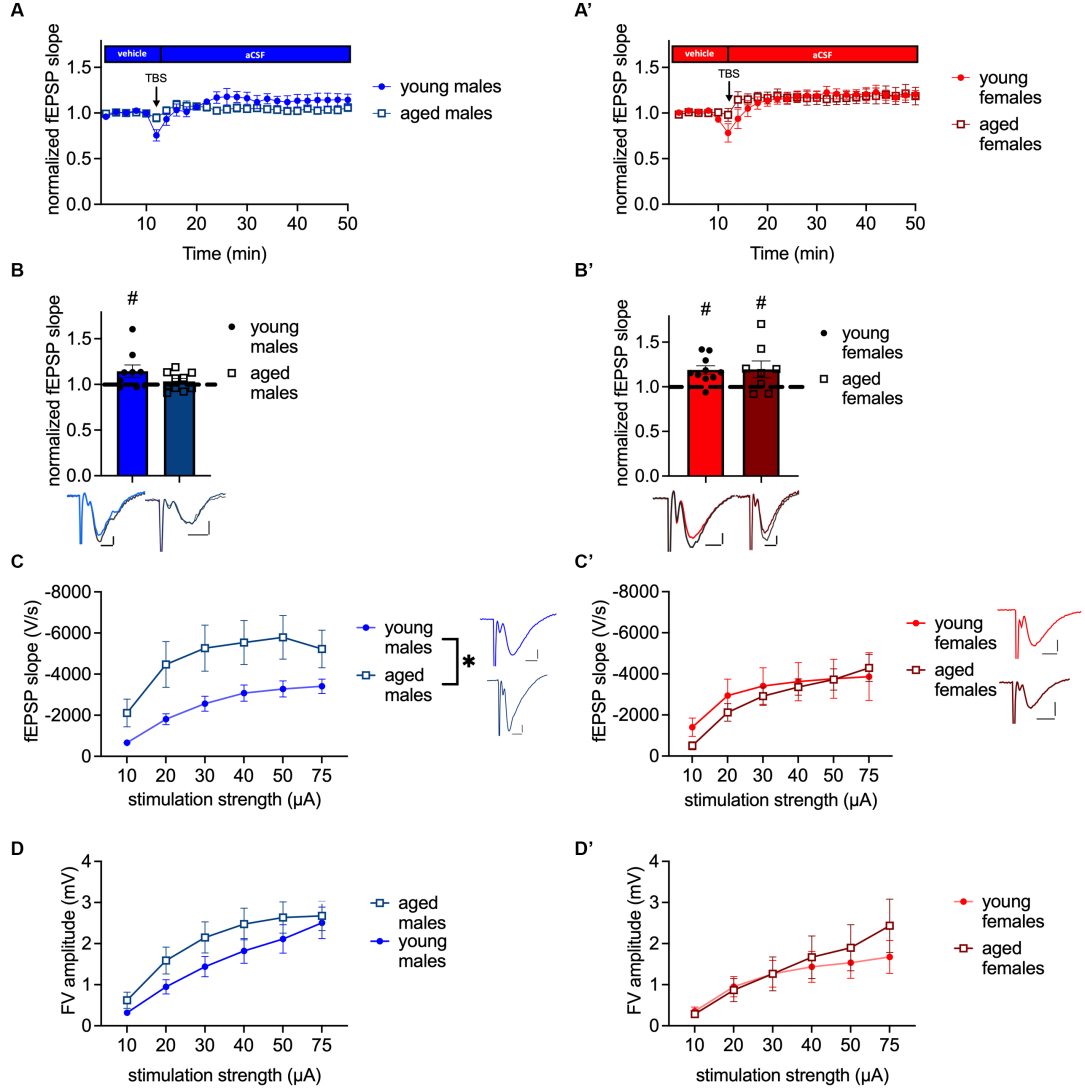
MPP-DG LTP is abolished in aged male mice, while baseline excitability is increased. **(A)** Timeline of MPP-DG LTP with the average values from young-adult and aged male mice. **(B)** Significant LTP is evident in young-adult but not aged males, as seen during the last 10 min of the recording. Representative fEPSP traces are plotted beneath. (pre-TBS colored, post-TBS gray). **(C)** Post-synaptic excitability is increased in aged male mice compared to young-adult males. Representative fEPSP traces are plotted at 30 μA stimulation strength. Scale bar *x*-axis 2 ms each and scale bar *y*-axis: 1 mV each. **(D)** Pre-synaptic excitability is not significantly altered in aged males. **(A′)** Timeline of MPP-DG LTP with the average values from young-adult and aged female mice **(B′)** Significant LTP is evident in both young-adult and aged female mice, as seen during the last 10 min of the recording. Representative fEPSP traces are plotted beneath (pre-TBS colored, post-TBS gray). **(C′)** Post-synaptic excitability is unaltered between young-adult and aged females. Representative fEPSP traces are plotted for young-adult and aged animals at 30 μA stimulation strength. Scale bar *x*-axis: 2 ms each and *y*-axis: 1 mV. **(D′)** Pre-synaptic excitability is not significantly altered in aged females. DMSO, dimethylsulfoxid. Age effect (group comparison): *p < 0.05; MPP-DG LTP induction (within slice comparison): #*p* < 0.05.

Since a decline of the NPYergic system has been related to memory impairment (Spiegel et al., 2013) we evaluated LTP at the MPP-DG synapse as the cellular correlate of memory (Bliss and Lomo, 1973) in both sexes and ages. Four repetitions of a TBS train, which was specifically designed to potentiate the electrical response of a synapse, were applied to reliably induce LTP under intact inhibition in young-adult males (*t*_(8)_ = 2.056, *p* = 0.0369, paired *t*-test, one-tailed, *n* = 9; Figures 2A,B) and females (*p* = 0.0186, Wilcoxon test, one-tailed, *n* = 10; Figures 2A′,B′), shown by an increased fEPSP slope.

A general impairment of LTP induction and maintenance in the DG *in vitro* of middle-aged rodents has previously been shown by Schreurs and colleagues (2017) using micro-array recordings (Schreurs et al., 2017), but changes of synaptic plasticity properties of aged female mice in the dDG have so far not been investigated. Our data confirmed that LTP is abolished in aged male mice under intact inhibition (t_(9)_ = 0.9207, *p* = 0.1906, paired t-test, one-tailed, *n* = 10; Figures 2A,B), as shown by the unchanged fEPSP slope after 4x TBS. By contrast, 4x TBS induced LTP successfully at the MPP-DG synapse of aged female mice (*t*_(7)_ = 2.019, *p* = 0.0416, paired *t*-test, one-tailed, *n* = 8; Figures 2A′,B′).

Potential aging-related changes in circuit functions were further assessed by evaluating post-synaptic and pre-synaptic excitability. Analysis of I-O curves revealed an increase in post-synaptic excitability in aged male mice in comparison to young-adult male mice (age: *F*_(1, 35)_ = 4.243, *p* = 0.0469; interaction: *F*_(5, 175)_ = 3.032, *p* = 0.0119, repeated-2-way ANOVA; Figure 2C). The post-hoc comparison showed that excitability was increased at 10, 20, 30, 40, and 50 μA (post-hoc comparison: 10 μA: *p* = 0.0476; 20 μA: *p* = 0.0302; 30 μA: *p* = 0.0313; 40 μA: *p* = 0.0405; and 50 μA: *p* = 0.0360, Fisher’s LSD, *n* = 20 (aged) and n = 17 (young-adult); Figure 2C). No change was observed in the pre-synaptic fiber volley (FV) amplitude of the aged male mice (*F*_(1, 34)_ = 1.377, *p* = 0.2487, repeated 2-way ANOVA, *n* = 20 (aged) and n = 16 (young-adult); Figure 2D). In contrast to males, post-synaptic excitability was unaltered in aged females compared to young-adult females (age: *F*_(1, 15)_ = 0.1063, *p* = 0.7489, interaction: *F*_(5, 75)_ = 1.261, *p* = 0.2899, repeated two-way ANOVA; *n* = 10 (young-adult females); *n* = 7 (aged females); Figure 2C′). Furthermore, the pre-synaptic excitability was unchanged in the aged females (age: *F*_(1, 14)_ = 0.1623, *p* = 0.6932, interaction: *F*_(5, 70)_ = 1.866, *p* = 0.1114, repeated two-way ANOVA; *n* = 9 (young-adult females) *n* = 7 (aged females); Figure 2D′). In summary, LTP in aged male mice is abolished, while it persists in aged females. Age-meditated LTP sex differences are accompanied by increased post-synaptic excitability in males, while post-synaptic excitability is unchanged during aging in females.

### 3.3. Cholinergic activation rescues MPP-DG LTP of aged male mice in a Y1-R-dependent manner

Based on our biochemical and physiological findings the potential involvement of the NPYergic system in aging-related synaptic functionality changes was further investigated in male mice.

**Figure 3:**
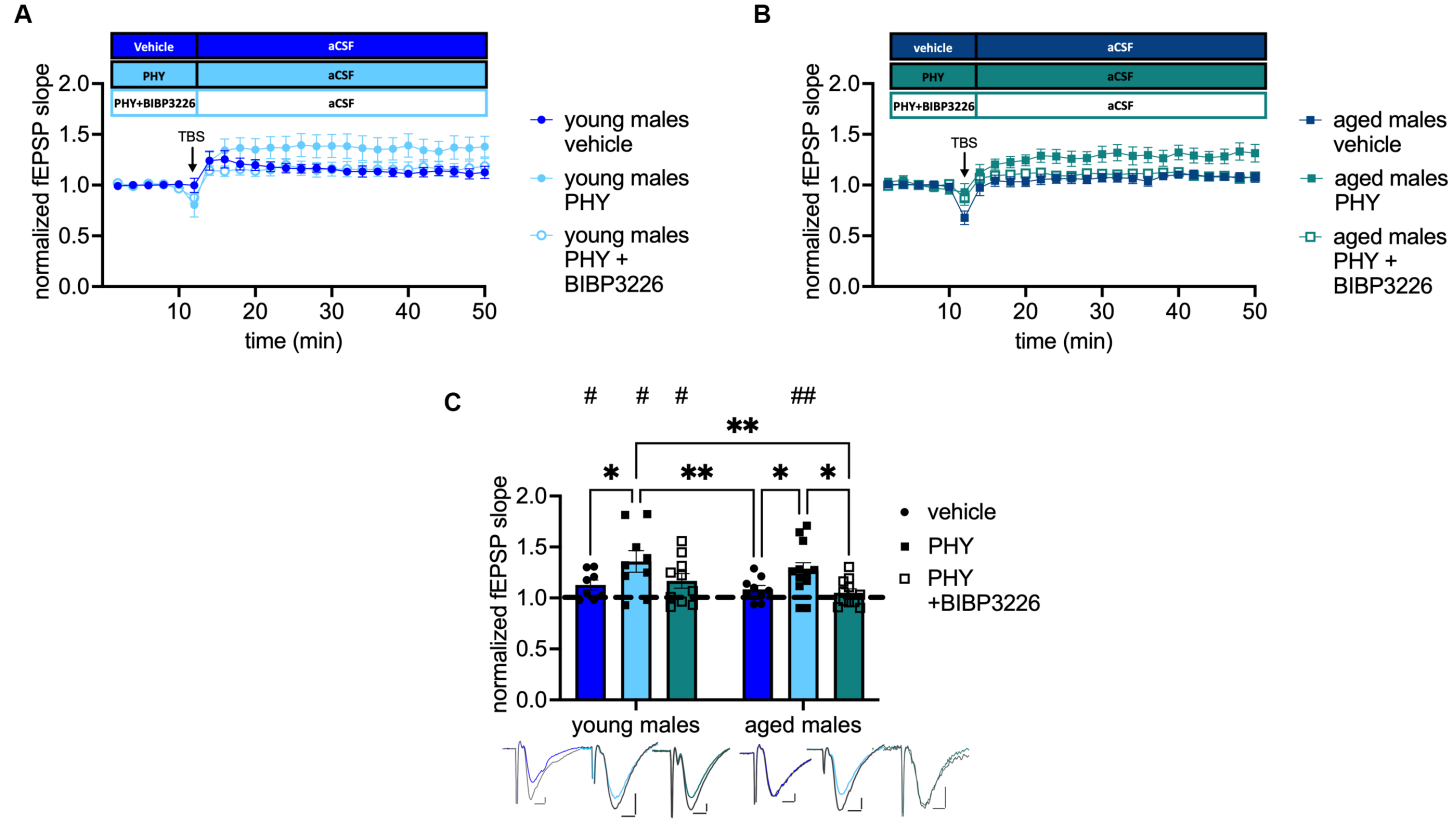
Cholinergic rescue of MPP-DG LTP in aged male mice is Y1-R-dependent. **(A)** Timeline of recordings from young-adult males with and without physostigmine (PHY) and Y1-R antagonist BIBP3226. **(B)** Corresponding timeline from aged males. **(C)** Physostigmine enhances LTP in young-adult males and reinstates it in aged males. In aged males, LTP is entirely prevented by the co-administration of BIBP3226. Representative fEPSP traces are plotted beneath (pre-TBS colored, post-TBS gray). Scale bar *x*-axis: 2 ms each and *y*-axis: 1 mV (young-adult males) and 0.4 mV (aged males). Changes in LTP strength (group comparison): *p < 0.05, **p < 0.01; LTP induction (within slice comparison): #*p* < 0.05, ##*p* < 0.01.

The beneficial effect of AChE inhibitor for AD treatment and on CA1 LTP in aged rodents is well documented (Yiannopoulou and Papageorgiou, 2020). By contrast, the beneficial effect of the cholinergic treatment on MPP-DG LTP and the involved mechanism have not been fully resolved yet. Based on our laboratory’s investigation that the ACh-mediated release of NPY is essential for memory retrieval in the DG of young-adult male mice (Raza et al., 2017), we evaluated the importance of Y1-R activation after cholinergic activation on MPP-DG LTP by applying the AChE inhibitor physostigmine (PHY 2 μM) and the combination of PHY and the selective Y1-R antagonist BIBP3226 (1 μM). Under cholinergic activation in young-adult male mice, LTP was facilitated compared to that in controls, regardless of Y1-R blockage (control: *t*_(7)_ = 2.469, *p* = 0.0214, paired *t*-test, one-tailed, *n* = 8; PHY: *t*_(8)_ = 2.403, *p* = 0.0215, paired *t*-test, one-tailed, *n* = 9; PHY + BIBP3226: *t*_(9)_ = 2.265, *p* = 0.0249, paired *t*-test, one-tailed, *n* = 10; Figure 3C). Furthermore, cholinergic activation by PHY rescued the LTP in aged male mice (control: *t*_(7)_ = 1.608, *p* = 0.0645, Wilcoxon test, one-tailed, *n* = 9; PHY: *t*_(12)_ = 3.821, *p* = 0.0012, paired t-test, one-tailed, *n* = 13; Figure 3C). However, this rescue was dependent on the activation of the Y1-R as shown by its abolition upon additional BIBP3226 application (*t*_(10)_ = 1.569, *p* = 0.0739, paired t-test, one-tailed, *n* = 11; Figure 3C). A treatment effect was depicted (*F*_(2, 53)_ = 6.338, *p* = 0.0034, two-way ANOVA; Figure 3C) by the increased LTP strength after PHY application in young-adult males (*p* = 0.0300, Fisher’s LSD test; Figure 3C) also compared to the LTP in aged male mice (control: *p* = 0.0085; PHY+ BIBP3226: *p* = 0.0022, Fisher’s LSD test; Figure 3C). Also, in aged mice LTP strength was enhanced by PHY compared to control (*p* = 0.0500, Fisher’s LSD test; Figure 3C) and significantly decreased after additional Y1-R blockade (*p* = 0.0158, Fisher’s LSD test; Figure 3C).

To note, blockade of Y1-R alone did not affect MPP-DG LTP induction in young-adult nor in aged animals (see Supplementary Figure A.1B). Nevertheless, Y1-R blockade alone led to significant differences in LTP strength between the groups (see Supplementary Figure A.1B). These results indicate a crucial role of Y1-R activation under moderate cholinergic activation in the MPP-DG LTP of aged male mice.

### 3.4. Decrease of baseline transmission upon pharmacological cholinergic activation depends on Y1-R activation in young-adult but not in aged male mice

**Figure 4:**
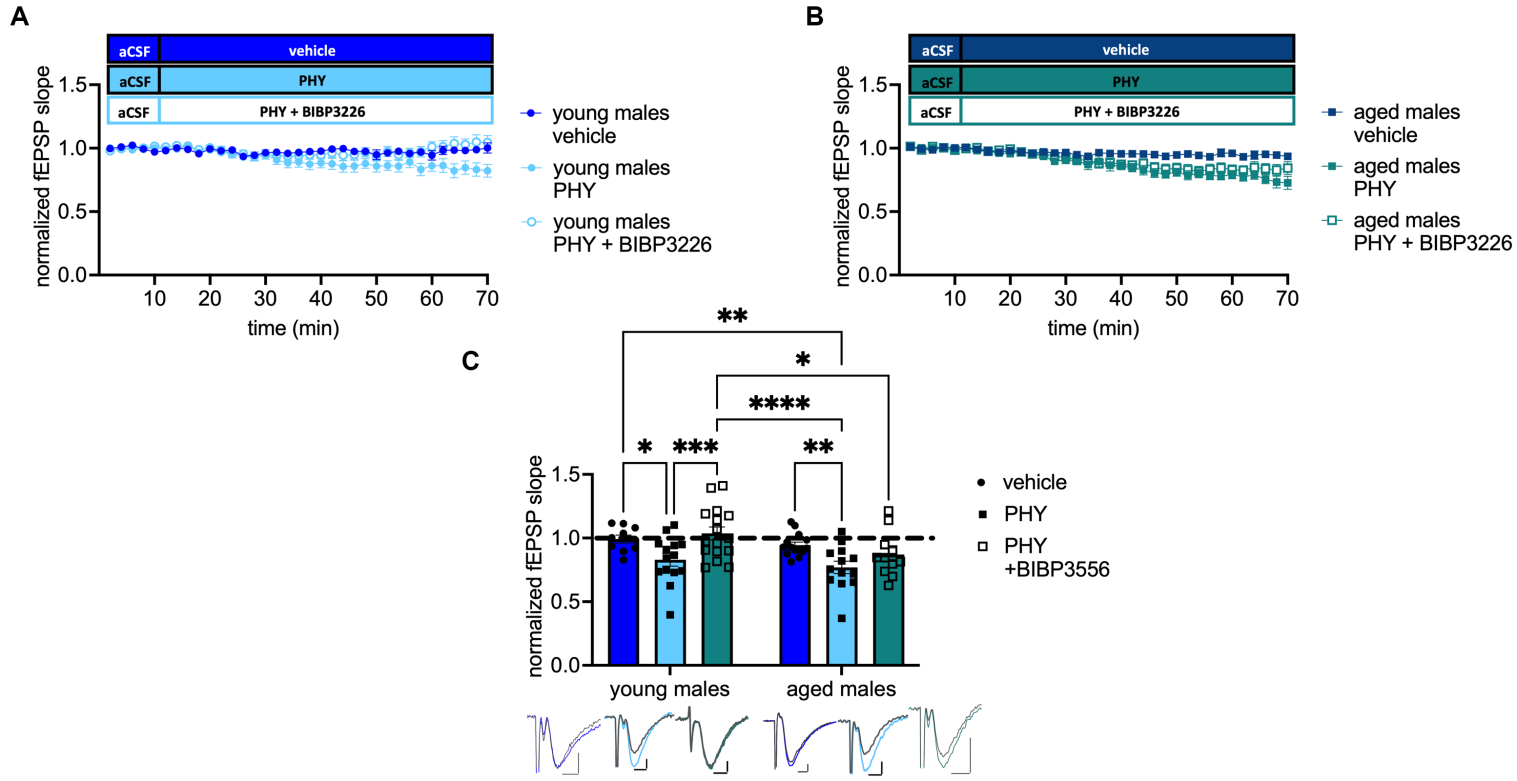
Pharmacological cholinergic activation promotes inhibition of excitability in a Y1-R and age-dependent manner. **(A)** Timeline of baseline transmission recordings from young-adult males with and without PHY and the Y1 antagonist BIBP3226. **(B)** Corresponding timeline from aged males. **(C)** PHY decreases baseline transmission in a Y1-R-dependent manner in young-adult but not aged males. Representative fEPSP traces are plotted beneath. Scale bar *x*-axis: 2 ms each and *y*-axis: 1 mV (young-adult mice control); 0.5 mV (aged mice PHY + BIBP3226) and 0.4 mV (young-adult and aged mice PHY, PHY + BIBP3226, and aged mice control; pre-application colored, post-application gray). Changes in fEPSP slope after Y1-R blockade (group comparison) *p < 0.05, **p < 0.01, ***p < 0.001, ****p < 0.0001.

Next, we tested the impact of moderate cholinergic activation on the Y1-R activation during neurotransmission. Strikingly, treatment (*F*_(2, 72)_ = 8.556 *p* = 0.0005, two-way ANOVA; Figure 4C) and age effects (*F*_(1, 72)_ = 5.918 *p* = 0.0175, two-way ANOVA; Figure 4C) were evident. Baseline transmission decreased after PHY application in young-adult (*post-hoc* comparison to young-adult males: *p* = 0.0186, Fisher’s LSD test, *n* = 10 (young-adult males), *n* = 14 (young-adult males + PHY); Figure 4C) and in aged male mice (*post-hoc* comparison to aged males: *p* = 0.0068, Fisher’s LSD test, *n* = 14 (aged males), *n* = 13 (aged males + PHY); Figure 4C). Strikingly, additional blockade of the Y1-R in young-adult male mice increased neurotransmission back to baseline (post-hoc comparison to: young-adult male mice + PHY: *p* = 0.0009; aged male mice + PHY: *p* < 0.000; *n* = 16 (young-adult males + PHY + BIBP3226), *n* = 13 (aged males + PHY); Figure 4C). However, this reversal was not observed in the aged males, as evident from the unchanged fEPSP slope (post-hoc comparison to aged males mice + PHY: *p* = 0.1353, Fisher’s LSD test, *n* = 11 (aged males + PHY + BIBP3226); *n* = 13 (aged males + PHY); Figure 4C) which was also not significant when compared to young-adult male mice + PHY (post-hoc comparison: *p* = 0.5316, Fisher’s LSD test, *n* = 11 (aged males PHY + BIBP3226); *n* = 14 (young-adult males + PHY); Figure 4C). Moreover, fEPSP slope remained lower than in young-adult male mice + PHY + BIBP3226 (post-hoc comparison: *p* = 0.0113, Fisher’s LSD test, *n* = 11 (aged males PHY + BIBP3226); *n* = 16 (young-adult males + PHY + BIBP3226); Figure 4C). An interaction (*F*_(2, 72)_ = 1.023 *p* = 0.3642, two-way ANOVA; Figure 4C) was not evident. To note, MPP-DG neurotransmission remained unchanged after selective Y1-R blockade independent of age, evident by the unchanged fEPSP slope (see Supplementary Figure A.2C). Nevertheless, an interaction effect reveals a significant increase of fEPSP slope in young-adult males treated with BIBP3226 compared to the corresponding aged group (see Supplementary Figure A.2C).

Taken together, increased cholinergic tonus mediated a decrease of MPP-DG neurotransmission in male mice in both young-adult and aged male mice. In contrast, the reversal of by Y1-R blockade was age-dependent and moderate cholinergic activation mediated Y1-R activation specifically in young-adult animals.

### 3.5. Application of NPY rescues LTP in aged male mice

**Figure 5:**
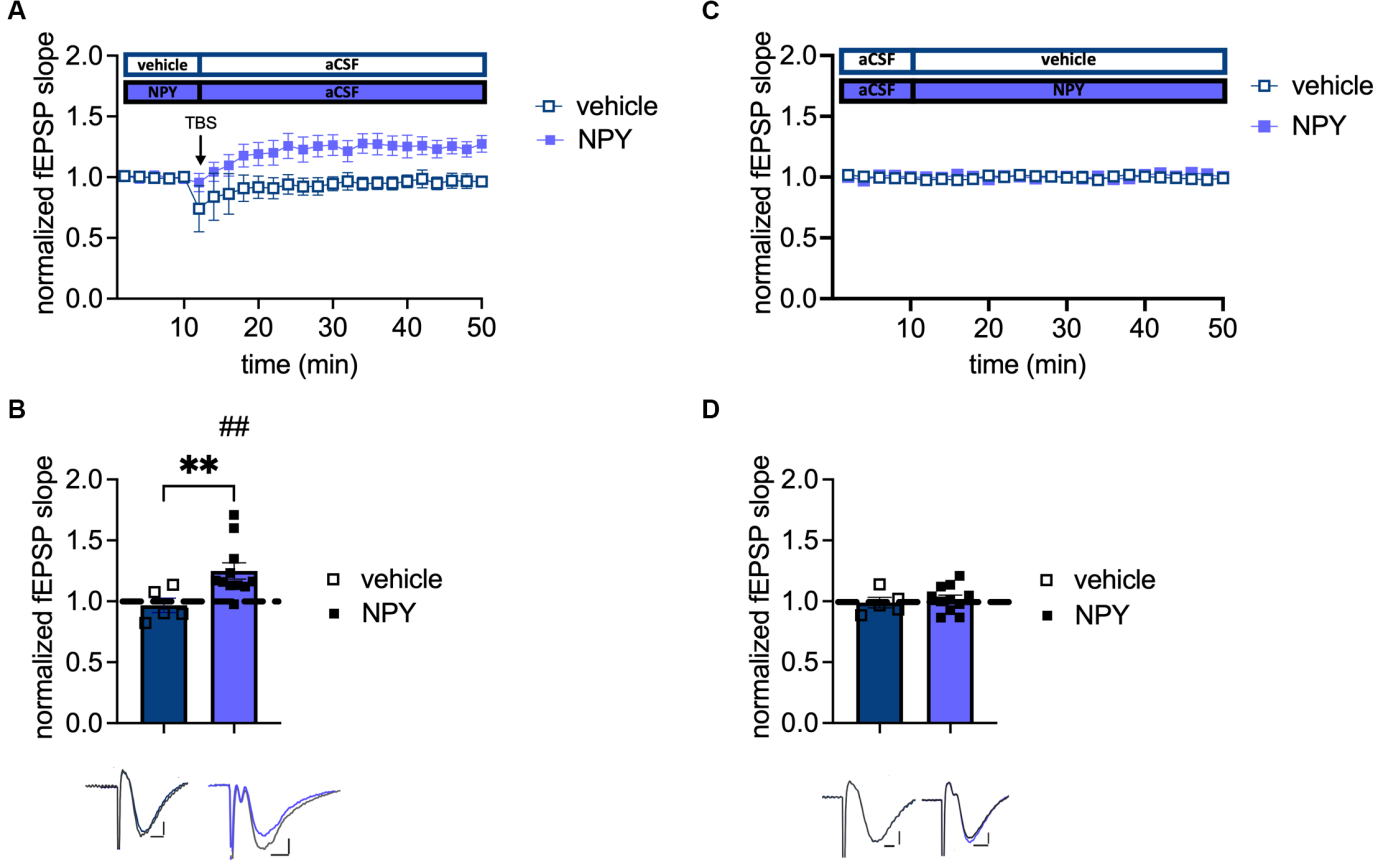
Increased NPYergic neurotransmission rescues LTP in aged male mice. **(A)** Timeline of plasticity recordings from aged males with and without exogenous NPY. **(B)** Recordings under vehicle conditions confirm the loss of LTP in aged male mice, while NPY reinstates a robust LTP in aged males. **(C)** Timeline of baseline transmission recordings from aged males with and without exogenous NPY. **(D)** Exogenous NPY application does not impact neurotransmission in aged male mice. Representative fEPSP traces are plotted beneath (pre-TBS/drug application colored and post-TBS/after drug application gray). Scale bar *x*-axis: 2 ms each and scale bar *y*-axis: 0.4 mV (control LTP) and 1 mV (transmission and NPY LTP). DMSO, dimethylsulfoxide. MPP-DG LTP strength (group comparison): **p < 0.01 and MPP-dDG LTP induction (in slice comparison): ##*p* < 0.01.

The data above indicate a crucial role of Y1-R activation to maintain plasticity during aging. Furthermore, Santos and colleagues conducted a beneficial effect of intracerebroventricular NPY infusion on spatial memory in young male mice (dos Santos et al., 2013). Therefore, the potential impact of NPY (1 μM) on plasticity in aged male mice was investigated. Application of NPY rescued LTP (t_(10)_ = 3.222, *p* = 0.0046, paired t-test, one-tailed, *n* = 11; Figure 5B) as evidenced by the increased fEPSP slope after TBS under NPY. Furthermore, NPY application led to a significantly increased LTP compared to vehicle controls (*p* = 0.0087, Mann–Whitney test, two-tailed, *n* = 11 (NPY); *n* = 5 (control); Figure 5B). By contrast, baseline neurotransmission was unchanged after NPY application (*t*_(14)_ = 0.5243, *p* = 0.6083, unpaired t-test, two-tailed, *n* = 5 (control), *n* = 11 (NPY); Figure 5D). This experiment underlines the importance of NPYergic tonus for successful LTP induction in aged male mice.

## 4. Discussion

In this study, we demonstrate a critical role of NPY in the loss of synaptic plasticity in the aged mouse DG and its recovery through stimulation of cholinergic neuromodulation. These data are of particular relevance considering the reported loss of NPYergic interneurons in the aged DG and reflected in aging-induced changes in NPY content of the structure, specifically in male mice.

To investigate the involvement of NPYergic interneurons in age-related changes of DG plasticity, we established slice preparation protocols for induction of LTP at the MPP-DG synapse under intact inhibition. These protocols produced a mild but reliable potentiation that are comparable to previous studies under intact GABAergic tonus (Snyder et al., 2001; Lopez-Rojas et al., 2016). Thereby we were able to demonstrate the expected loss of LTP in aged male mice and its recovery upon cholinergic stimulation, which is in line with various *in vivo* studies reporting impaired LTP at the PP-DG synapse of aged male rodents (Barnes, 1979; Geinisman et al., 1992). By contrast we found that LTP was maintained in the DG of naturally aged females even in the absence of an AChE inhibitor, in line with findings in a female Alzheimer’s diseases mouse model (Yun et al., 2007).

Given the high but age-dependent decaying expression of NPY in the male DG and the constant low levels of this peptide in females, we tested a possible involvement of the peptide in the observed plasticity effects of male mice. In fact, we observed that recovery of LTP upon cholinergic stimulation in aged animals was dependent on NPY-Y1 receptors, the major post-synaptic NPY receptor type in the DG (Figure 3C). By contrast, the reduction of baseline transmission induced by cholinergic stimulation persisted under NPY-Y1 receptor blockage in the aged animals, in contrast to young (Figure 4C). Therefore, NPY-release triggered by enhanced cholinergic transmission might underlie the reduced baseline transmission and enhanced LTP strength both in young and aged mice. However, their (LTP vs. synaptic transmission) dependency on the activation of Y1-R diverges across the ages. These observations suggest that NPY availability in the DG circuit is critical for determining Y1-R dependency of LTP vs. synaptic transmission: (1) In aged mice, probably due to already reduced NPY levels, in addition to cholinergic stimulation, a rather strong stimulation paradigm (e.g., TBS) is required to recruit a critical number of NPY-releasing neurons, thus, NPY release for sufficient activation of Y1-R in aged male mice. (2) In young mice, NPY release triggered via constitutive cholinergic activation under baseline conditions seems to be sufficient to act on Y1-R and exert an effect. (3) In young mice, during LTP induction via TBS under constitutive cholinergic activation, additional Y1-R independent mechanisms seem to be involved which need to be elucidated in further research. Moreover, unchanged pre-synaptic response in aged male mice (Figure 2D) indicates that MPP-DG LTP deficits were related to local changes in the DG during aging rather than alterations of baseline excitability or a non-functional input from the EC (Geinisman et al., 1992; Scheff et al., 2006).

A decline of the projections from the MS to the DG during aging has been previously described (Ballinger et al., 2016). These cholinergic projections from the MS target both GCs and hilar interneurons, including NPY/SST-containing HIPP cells and parvalbumin-positive basket cells, and axo-axonic cells (Ogando et al., 2021). HIPP cells are vulnerable to a cholinergic decline (Matsuoka et al., 1995) and mediate cholinergic stimulation of NPY-Y1 receptors at the DG GCs (Raza et al., 2017). Non-fast spiking interneurons, including HIPP cells, are strongly recruited after strong PP activation to maintain the quiescence of GC (Liu et al., 2014) and NPY is known to reduce glutamatergic transmission and the release of Ca^2+^ in GCs (Sperk et al., 2007). Moreover, a reconfiguration effect of cholinergic transmission on the hilar interneuron network, particularly SST^+^ (and putatively NPY^+^) cells and PV+ basket cells, has been reported (Ogando et al., 2021) that might mediate the facilitatory effect of muscarinic transmission on LTP at MPP-DG synapses (Abe et al., 1994; Ogando et al., 2021). Intriguingly, a subpopulation of PV+ basket cells and axo-axonic cells in the hilus also expresses NPY (Comeras et al., 2021). It is thus conceivable that enhanced cholinergic tonus might act in part by stimulating NPY release from residual hilar interneurons in the aged animals to overcome the aging-related local deficit in this peptide (Figure 1B). In support of this explanation, we could show that application of NPY alone, in the absence of acetylcholinesterase blockade was sufficient to reinstate the ability for induction of LTP (Figure 5B). Interestingly, both expression and physiological data indicate that NPY appears to play a sex-specific role in DG function during aging, more relevant to male than to female mice.

As mentioned above, under cholinergic activation, NPY concentration in the DG seems to be a critical factor for Y1-R dependency of LTP across the ages. Y1-R dependency of LTP is only evident in low NPY conditions (aged mice) but not when NPY levels are high (young mice). Considering the recruitment of Y1-R independent mechanisms under high NPY levels as observed in young mice (Figure 3C), we suggest that the LTP induced by exogenous NPY application in aged mice could possibly be Y1-R independent (Figures 5A,B). Therefore, there seems to be a delicate NPY-concentration dependent recruitment of Y1-R dependent vs. independent mechanisms across the ages which needs to be further investigated in the future.

**Figure 6:**
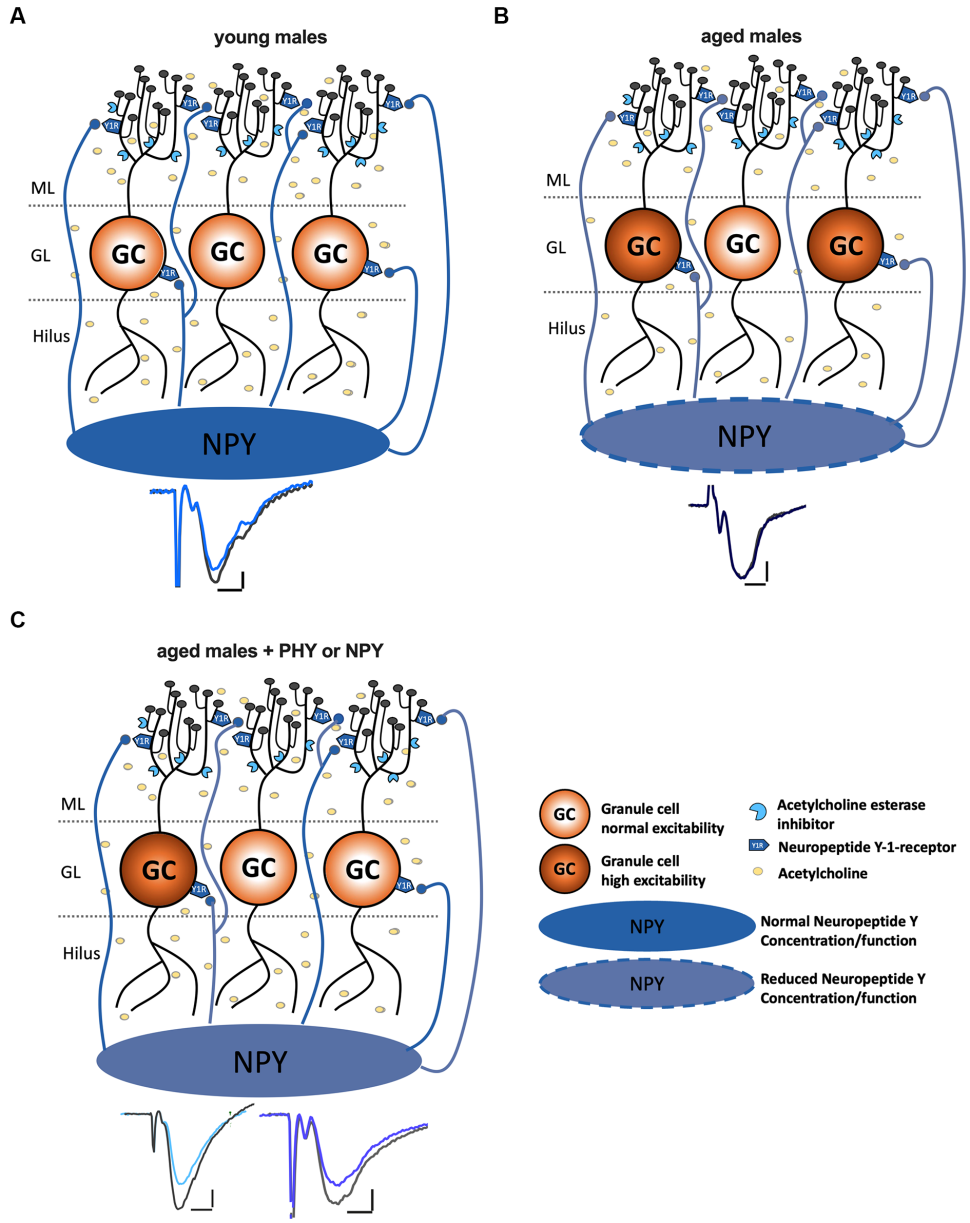
Increase of NPYergic tonus recovers LTP in aged male mice. **(A)** In young male mice LTP induction is successful under intact GABAergic inhibition with the TBS protocol. **(B)** In aged male mice, the concentration of NPY, as well as the cholinergic tonus, are decreased. The reduced NPY concentration might cause hyperexcitability of GCs and a shift in the E/I balance. **(C)** An increase in cholinergic activity or endogenous as well as exogenous NPY levels restores LTP in aged male mice potentially via normalizing GC hyperactivation and restoring E/I balance.

We considered the possibility that the effect of NPY may at least in part be mediated by recovering excitation-inhibition balance in the DG, as prominent post-synaptic hyperexcitability could be observed in the aged animals (Figure 2C). E-I balance is essential for maintaining signal-to-noise ratio, information capacity, and sparse GC activation in the DG (Marder and Buonomano, 2004; Shew et al., 2011). Aged rodent DG displays increased glutamate release (Saransaari, 1995; El-Hayek et al., 2013), a higher population spike to a given PP stimulus, and a reduced voltage threshold (Barnes and Mcnaughton, 1980). Moreover, increased E-I ratio in aged rats after LPP stimulation correlates with reduced cognitive performance (Tran et al., 2019) and enhanced post-synaptic inhibitory strength has been found in cognitively unimpaired-aged rats (Tran et al., 2018). NPY Y1-receptor activation might be able to reduce glutamate release and Ca^2+^ influx and, with that, reduce GC excitability (Sperk et al., 2007). However, the application of NPY in our experiments did not affect MPP-DG transmission (Figure 5D), and NPY-Y1 receptor blockage failed to interfere with the cholinergic reduction of synaptic transmission in aged mice (Figure 4C). This is in line with previous studies in young-adult animals showing no or minor effects of NPY on PP-evoked EPSPs in the molecular layer (Sperk et al., 2007). Thus, in aged mice, NPYergic transmission appears to become particularly relevant for the induction of plasticity in the MPP-DG pathway (Figure 6).

Various studies have demonstrated a role of NPY and NPY Y1 receptors in emotional and learning deficits in Alzheimer’s disease models and their cholinergic recovery (dos Santos et al., 2013; Borroto-Escuela et al., 2022). However, a memory-enhancing effect of NPY overexpression in the hippocampus and hypothalamus of rats is not observed in middle-aged animals, while its anxiolytic-like effects persist (Carvajal et al., 2004). Given the prominent role of the DG interneurons in both cognition and emotion (Hadad-Ophir et al., 2014; Tripathi et al., 2021; Albrecht et al., 2022), NPY appears to act as a network modulator that integrates, in a sex-dependent manner, the response of the hilar network to stress- and aging-induced alterations in excitability and plasticity and may lend itself as a target of intervention.

## Data availability statement

The raw data supporting the conclusions of this article will be made available by the authors, without undue reservation.

## Ethics statement

Ethical approval was not required for the study involving animals in accordance with the local legislation and institutional requirements because no approval is needed for *ex-vivo* experiments which are based on organ harvesting (in this case the brain of mice).

## Author contributions

KK:. Writing – original draft, Writing – review & editing, Conceptualization, Data curation, Formal analysis, Investigation, Visualization, Methodology. MÁ: Investigation, Writing – review & editing. GÇ: Writing – review & editing, Conceptualization, Methodology, Supervision. OS: Conceptualization, Funding acquisition, Methodology, Project administration, Resources, Supervision, Writing – review & editing.

## References

[ref1] Abe K. Nakata A. Mizutani A. Saito H. (1994). Pergamon 0028-3908(94)EOO34-0 Facilitatory but nonessential Role of the muscarinic cholinergic system in the generation of long-term potentiation of population spikes in the dentate gyrus in vivo. Neuropharmacology 33, 847–852. doi: 10.1016/0028-3908(94)90180-5, PMID: 7969803

[ref2] Albrecht A. Müller I. Weiglein A. Pollali E. Çalışkan G. Stork O. (2022). Choosing memory retrieval strategies: a critical role for inhibition in the dentate gyrus. Neurobiol. Stress 20:100474. doi: 10.1016/j.ynstr.2022.100474, PMID: 35958670 PMC9357949

[ref3] Amani M. Lauterborn J. C. le A. A. Cox B. M. Wang W. Quintanilla J.. (2021). Rapid aging in the perforant path projections to the rodent dentate gyrus. J. Neurosci. 41, 2301–2312. doi: 10.1523/JNEUROSCI.2376-20.2021, PMID: 33514675 PMC8018768

[ref4] Armstrong C. M. J. Conti F. Defelice J. Wanke E. (1982). N europeptide Y-a novel brain peptide with structural similarities to peptide YY and pancreatic polypeptide. J. Physiol. Lond. 296, 659–660. doi: 10.1038/296659a0 6896083

[ref5] Bakker A. Krauss G. L. Albert M. S. Speck C. L. Jones L. R. Stark C. E.. (2012). Reduction of hippocampal hyperactivity improves cognition in amnestic mild cognitive impairment. Neuron 74, 467–474. doi: 10.1016/j.neuron.2012.03.023, PMID: 22578498 PMC3351697

[ref6] Ballinger E. C. Ananth M. Talmage D. A. Role L. W. (2016). Basal forebrain cholinergic circuits and signaling in cognition and cognitive decline. Neuron 91, 1199–1218. doi: 10.1016/j.neuron.2016.09.006 27657448 PMC5036520

[ref7] Baratta M. V. Lamp T. Tallent M. K. (2002). Somatostatin depresses long-term potentiation and Ca2+ signaling in mouse dentate gyrus. J. Neurophysiol. 88, 3078–3086. doi: 10.1152/jn.00398.2002 12466431

[ref8] Barnes C. A. (1979). Memory deficits associated with Senescence: a neurophysiological and Behavioral study in the rat. J. Comp. Physiol. Psychol. 93, 74–104. doi: 10.1037/h0077579, PMID: 221551

[ref9] Barnes C. A. Mcnaughton B. L. (1980). Physiological compensation for loss of afferent synapses in rat hippocampal granule cells during senescence. J. Physiol. 309, 473–485. doi: 10.1113/jphysiol.1980.sp013521, PMID: 7252877 PMC1274597

[ref10] Berger-Sweeney J. Stearns N. A. Murg S. L. Floerke-Nashner L. R. Lapp D. A. Baxter A. G. (2001). Selective immunolesions of cholinergic neurons in mice: effects on neuroanatomy, neurochemistry, and behavior. J. Neurosci. 21, 8164–8173. 11588189 10.1523/JNEUROSCI.21-20-08164.2001 PMC6763842

[ref11] Bliss T. V. P. Lomo T. (1973). Long-lasting potentiation of synaptic transmission in the dentate area of the anaesthetized rabbit following stimulation of the perforant path. J. Physiol. 232, 357–374. doi: 10.1113/jphysiol.1973.sp010273, PMID: 4727084 PMC1350458

[ref12] Borroto-Escuela D. O. Fores R. Pita M. Barbancho M. A. Zamorano-Gonzalez P. Casares N. G.. (2022). Intranasal delivery of Galanin 2 and neuropeptide Y1 agonists enhanced spatial memory performance and neuronal precursor cells proliferation in the dorsal Hippocampus in rats. Front. Pharmacol. 13:820210. doi: 10.3389/fphar.2022.820210, PMID: 35250569 PMC8893223

[ref13] Botelho M. Cavadas C. (2015). Neuropeptide Y: an anti-aging player? Trends Neurosci. 38, 701–711. doi: 10.1016/j.tins.2015.08.012 26549884

[ref14] Carvajal C. C. Vercauteren F. Dumont Y. Michalkiewicz M. Quirion R. (2004). Aged neuropeptide Y transgenic rats are resistant to acute stress but maintain spatial and non-spatial learning. Behav. Brain Res. 153, 471–480. doi: 10.1016/j.bbr.2004.01.004, PMID: 15265645

[ref15] Comeras L. B. Hörmer N. Mohan Bethuraj P. Tasan R. O. (2021). NPY released from GABA neurons of the dentate gyrus specially reduces contextual fear without affecting cued or trace fear. Front. Synap. Neurosci. 13:635726. doi: 10.3389/fnsyn.2021.635726, PMID: 34122036 PMC8187774

[ref16] dos Santos V. V. Santos D. B. Lach G. Rodrigues A. L. S. Farina M. de Lima T. C. M.. (2013). Neuropeptide Y (NPY) prevents depressive-like behavior, spatial memory deficits and oxidative stress following amyloid-β (Aβ1-40) administration in mice. Behav. Brain Res. 244, 107–115. doi: 10.1016/j.bbr.2013.01.039, PMID: 23396168

[ref17] El-Hayek Y. H. Wu C. Ye H. Wang J. Carlen P. L. Zhang L. (2013). Hippocampal excitability is increased in aged mice. Exp. Neurol. 247, 710–719. doi: 10.1016/j.expneurol.2013.03.012 23510762

[ref18] Figueroa A. G. Benkwitz C. Surges G. Kunz N. Homanics G. E. Pearce R. A. (2021). Hippocampal β2-GABAA receptors mediate LTP suppression by etomidate and contribute to long-lasting feedback but not feedforward inhibition of pyramidal neurons. J. Neurophysiol. 126, 1090–1100. doi: 10.1152/jn.00303.2021, PMID: 34406874 PMC8560413

[ref19] Freund T. F. Buzsáki G. (1996). Interneurons of the Hippocampus. Hippocampus 6, 347–470. 8915675 10.1002/(SICI)1098-1063(1996)6:4<347::AID-HIPO1>3.0.CO;2-I

[ref20] Froc D. J. Eadie B. Li A. M. Wodtke K. Tse M. Christie B. R. (2003). Reduced synaptic plasticity in the lateral perforant path input to the dentate gyrus of aged C57BL/6 mice. J. Neurophysiol. 90, 32–38. doi: 10.1152/jn.00105.2003 12634277

[ref21] Geinisman Y. Detoledo-Morrell L. Morrell F. (1992). Comparison of Structural synaptic modifications induced by long-term potentiation in the hippocampal dentate gyrus of young adult and aged rats. Ann. N. Y. Acd. Sci. 15, 452–466. doi: 10.1111/j.1749-6632.1994.tb44428.x 7847690

[ref27] Geinisman Y. I. Detoledo-Morrell L. Morrellt F. Heller R. E. (1995). Hippocampal markers of age-related memory dysfunction: behavioral, electrophysiological and morphological perspectives. Progr. Neurobiol. 45. doi: 10.1016/0301-0082(94)00047-l 7777673

[ref22] Gilbert M. E. Mundy W. R. Crofton K. M. (2000). Spatial learning and long-term potentiation in the dentate gyrus of the Hippocampus in animals developmentally exposed to Aroclor 1254. Toxicol. Sci. 57, 102–111. doi: 10.1093/toxsci/57.1.102 10966516

[ref23] Hadad-Ophir O. Albrecht A. Stork O. Richter-Levin G. (2014). Amygdala activation and GABAergic gene expression in hippocampal sub-regions at the interplay of stress and spatial learning. Front. Behav. Neurosci. 8:e00003. doi: 10.3389/fnbeh.2014.00003, PMID: 24478650 PMC3896990

[ref24] Hagihara H. Toyama K. Yamasaki N. Miyakawa T. (2010). Dissection of hippocampal dentate gyrus from adult mouse. J. Vis. Exp. doi: 10.3791/1543, PMID: 19920804 PMC3142893

[ref25] Hattiangady B. Rao M. S. Shetty G. A. Shetty A. K. (2005). Brain-derived neurotrophic factor, phosphorylated cyclic AMP response element binding protein and neuropeptide Y decline as early as middle age in the dentate gyrus and CA1 and CA3 subfields of the hippocampus. Exp. Neurol. 195, 353–371. doi: 10.1016/j.expneurol.2005.05.014, PMID: 16002067

[ref26] Houser C. R. (2007). Interneurons of the dentate gyrus: an overview of cell types, terminal fields and neurochemical identity. Prog. Brain Res. 163:63013. doi: 10.1016/S0079-6123 17765721

[ref28] Kesner R. P. Crutcher K. A. Measom M. O. (1986). Medial septal and nucleus basalis Magnocellularis lesions produce order memory deficits in rats which mimic symptomatology of Alzheimer’s disease. Neurobiol. Aging 7, 287–295. doi: 10.1016/0197-4580(86)90009-6 3528890

[ref29] Liu Y. C. Cheng J. K. Lien C. C. (2014). Rapid dynamic changes of dendritic inhibition in the dentate gyrus by presynaptic activity patterns. J. Neurosci. 34, 1344–1357. doi: 10.1523/JNEUROSCI.2566-13.2014, PMID: 24453325 PMC6705299

[ref30] Lopez-Rojas J. Heine M. Kreutz M. R. (2016). Plasticity of intrinsic excitability in mature granule cells of the dentate gyrus. Sci. Rep. 6, 1–12. doi: 10.1038/srep21615 26857841 PMC4746665

[ref31] Lukoyanov N. V. Andrade J. Â. P. Madeira M. D. Paula-Barbosa M. M. (1999). Effects of age and sex on the water maze performance and hippocampal cholinergic ®bers in rats. Comp. Stud. 269, 141–144. doi: 10.1016/S0304-3940(99)00442-5 10454152

[ref32] Madronal N. Delgado-García J. M. Fernándes-Guizán A. Chatterjee J. Köhn M. Mattucci C.. (2016). Rapid erasure of hippocampal memory following inhibition of dentate gyrus granule cells. Nat. Commun. 7:10923. doi: 10.1038/ncomms10923, PMID: 26988806 PMC4802048

[ref33] Marder C. P. Buonomano D. V. (2004). Timing and balance of inhibition enhance the effect of long-term potentiation on cell firing. J. Neurosci. 24, 8873–8884. doi: 10.1523/JNEUROSCI.2661-04.2004 15470154 PMC6729972

[ref34] Matsuoka N. Yamazaki M. Yamaguchi I. (1995). Changes in brain somatostatin in memory-deficient rats: comparison with cholinergic markers. Neuroscience 66, 617–626. doi: 10.1016/0306-4522(94)00628-I 7644025

[ref35] Nahvi R. J. Sabban E. L. (2020). Sex differences in the neuropeptide Y system and implications for stress related disorders. Biomolecules 10, 1–21. doi: 10.3390/biom10091248 PMC7564266 32867327

[ref36] Ogando M. B. Pedroncini O. Federman N. Romano S. A. Brum L. A. Lanuza G. M.. (2021). Cholinergic modulation of dentate gyrus processing through dynamic reconfiguration of inhibitory circuits. Cell Rep. 36:109572. doi: 10.1016/j.celrep.2021.109572, PMID: 34433032

[ref37] Pang K. Williams M. J. Olton D. S. (1993). Activation of the medial septal area attenuates LTP of the lateral perforant path and enhances heterosynaptic LTD of the medial perforant path in aged rats. Brain Res. 632, 150–160. doi: 10.1016/0006-8993(93)91150-q, PMID: 8149224

[ref38] Ramirez S. Liu X. Lin P.-A. Suh J. Pignatelli M. Redondo R. L.. (2013). Creating a false memory in the hippocampus. Science 341, 387–391. doi: 10.1126/science.1239073 23888038

[ref39] Raza S. A. Albrecht A. Çalışkan G. Müller B. Demiray Y. E. Ludewig S.. (2017). HIPP neurons in the dentate gyrus mediate the cholinergic modulation of background context memory salience. Nat. Commun. 8:189. doi: 10.1038/s41467-017-00205-3, PMID: 28775269 PMC5543060

[ref40] Rodgers F. C. Zarnowska E. D. Laha K. T. Engin E. Zeller A. Keist R.. (2015). Etomidate impairs long-term potentiation in vitro by targeting α5-subunit containing GABAA receptors on nonpyramidal cells. J. Neurosci. 35, 9707–9716. doi: 10.1523/JNEUROSCI.0315-15.2015, PMID: 26134653 PMC4571505

[ref41] Saransaari P. (1995). ELSEVIER mechanisms of ageing and development and dl? wloplIMt age-related changes in the uptake and release of glutamate and aspartate in the mouse brain. Oja Mechan. Age. Dev. 81, 61–71. doi: 10.1016/0047-6374(95)01583-L 8569281

[ref42] Scheff S. W. Price D. A. Schmitt F. A. Mufson E. J. (2006). Hippocampal synaptic loss in early Alzheimer’s disease and mild cognitive impairment. Neurobiol. Aging 27, 1372–1384. doi: 10.1016/j.neurobiolaging.2005.09.012 16289476

[ref43] Schreurs A. Sabanov V. Balschun D. (2017). Distinct properties of long-term potentiation in the dentate gyrus along the Dorsoventral Axis: influence of age and inhibition. Sci. Rep. 7:5157. doi: 10.1038/s41598-017-05358-1, PMID: 28698637 PMC5506024

[ref44] Seo H. J. Park J. E. Choi S. M. Kim T. Cho S. H. Lee K. H.. (2021). Inhibitory neural network’s impairments at hippocampal CA1 LTP in an aged transgenic mouse model of Alzheimer’s disease. Int. J. Mol. Sci. 22, 1–15. doi: 10.3390/ijms22020698 PMC7828160 33445678

[ref45] Shew W. L. Yang H. Yu S. Roy R. Plenz D. (2011). Information capacity and transmission are maximized in balanced cortical networks with neuronal avalanches. J. Neurosci. 31, 55–63. doi: 10.1523/JNEUROSCI.4637-10.2011, PMID: 21209189 PMC3082868

[ref46] Snyder J. S. Kee N. Wojtowicz J. M. (2001). Effects of adult neurogenesis on synaptic plasticity in the rat dentate gyrus. J. Neurophysiol. 85, 2423–2431. doi: 10.1152/jn.2001.85.6.2423 11387388

[ref47] Sperk G. Hamilton T. Colmers W. F. (2007). Neuropeptide Y in the dentate gyrus. Prog. Brain Res. 163, 285–297. doi: 10.1016/S0079-6123(07)63017-9 17765725

[ref48] Spiegel A. M. Koh M. T. Vogt N. M. Rapp P. R. Gallagher M. (2013). Hilar interneuron vulnerability distinguishes aged rats with memory impairment. J. Comp. Neurol. 521, 3508–3523. doi: 10.1002/cne.23367, PMID: 23749483 PMC4801143

[ref49] Squire L. R. Zola S. M. (1996). Memory: recording experience in cells and circuits: diversity in memory research. Proc. Natl. Acad. Sci. U.S.A. 93, 13435–13437. doi: 10.1073/pnas.93.24.13435 PMC33627 8942953

[ref51] Tran T. Bridi M. Koh M. T. Gallagher M. Kirkwood A. (2019). Reduced cognitive performance in aged rats correlates with increased excitation/inhibition ratio in the dentate gyrus in response to lateral entorhinal input. Neurobiol. Aging 82, 120–127. doi: 10.1016/j.neurobiolaging.2019.07.010, PMID: 31476654 PMC9216165

[ref52] Tran T. Gallagher M. Kirkwood A. (2018). Enhanced postsynaptic inhibitory strength in hippocampal principal cells in high-performing aged rats. Neurobiol. Aging 70, 92–101. doi: 10.1016/j.neurobiolaging.2018.06.008, PMID: 30007169 PMC6432915

[ref53] Tripathi K. Demiray Y. E. Kliche S. Jing L. Hazra S. Hazra J. D.. (2021). Reducing glutamic acid decarboxylase in the dorsal dentate gyrus attenuates juvenile stress induced emotional and cognitive deficits. Neurobiol. Stress 15:100350. doi: 10.1016/j.ynstr.2021.100350, PMID: 34150959 PMC8193143

[ref54] Yassa M. A. Muftuler L. T. Stark C. E. L. (2010). Ultrahigh-resolution microstructural diffusion tensor imaging reveals perforant path degradation in aged humans in vivo. Proc. Natl. Acad. Sci. U. S. A. 107, 12687–12691. doi: 10.1073/pnas.1002113107, PMID: 20616040 PMC2906542

[ref55] Yiannopoulou K. G. Papageorgiou S. G. (2020). Current and future treatments in Alzheimer disease: an update. J. Cent. Nerv. Syst. Dis. 12:90739. doi: 10.1177/1179573520907397, PMID: 32165850 PMC7050025

[ref50] Yun S. H. Park K. A. Kwon S. Woolley C. S. Sullivan P. M. Pasternak J. F.. (2007). Estradiol enhances long term potentiation in hippocampal slices from aged ApoE4-TR mice. Hippocampus 17, 1153–1157. 17696167 10.1002/hipo.20357

